# Supplementary material for: The Janus Role of Adhesion in Chondrogenesis
Source: Int J Mol Sci. 2020 Jul 24;21(15):5269. doi: 10.3390/ijms21155269 (PMC7432906; doi:10.3390/ijms21155269)
Supplement: Supplementary file 1 [file ijms-21-05269-s001.pdf]

Supporting Information

The Janus role of adhesion in chondrogenesis

Ignasi Casanellas[1,2,3], Anna Lagunas[3,1,*], Yolanda Vida[4,5], Ezequiel Pérez-Inestrosa[4,5], José A. Andrades[6,3], José Becerra[6,3,5] and Josep Samitier[1,2,3]

[1] Institute for Bioengineering of Catalonia (IBEC), Barcelona Institute of Science and Technology (BIST), 08028 Barcelona, Spain; icasanellas@ibecbarcelona.eu (I.C.); alagunas@ibecbarcelona.eu (A.L.); samitier@ibecbarcelona.eu (J.S.)

[2] Department of Electronics and Biomedical Engineering, University of Barcelona (UB), 08028 Barcelona, Spain

[3] Networking Biomedical Research Center in Bioengineering, Biomaterials and Nanomedicine (CIBER-BBN), 28029 Madrid, Spain

[4] Universidad de Málaga-IBIMA, Departamento de Química Orgánica, 29071 Málaga, Spain ; yolvida@uma.es (Y.V.); inestrosa@uma.es (E.P.-I.)

[5] Centro Andaluz de Nanomedicina y Biotecnología-BIONAND, 29590 Campanillas, Málaga, Spain

[6] Department of Cell Biology, Genetics and Physiology, Universidad de Málaga-IBIMA, 29071 Málaga, Spain; andrades@uma.es (J.A.A.); becerra@uma.es (J.B.)

*Correspondence: alagunas@ibecbarcelona.eu

Supplementary Materials and Methods

Cell viability assay

Cells were cultured on nanopatterned or fibronectin-coated substrates as described in the Materials and Methods section. A commercial viability/cytotoxicity cell labelling kit was used for the assay (ThermoFisher, L3224). At day 7 of chondrogenesis, samples were washed twice with PBS and stained with calcein AM and ethidium homodimer-1 at 4 μM in non-serum containing medium for 40 min at 37°C and 5% CO_2. Samples were washed twice with PBS, mounted on microscopy slides, and immediately imaged in a Leica SPE Upright Confocal Microscope (Leica Microsystems) with a 40X objective. Z-projections of cell condensates were produced with Fiji software.

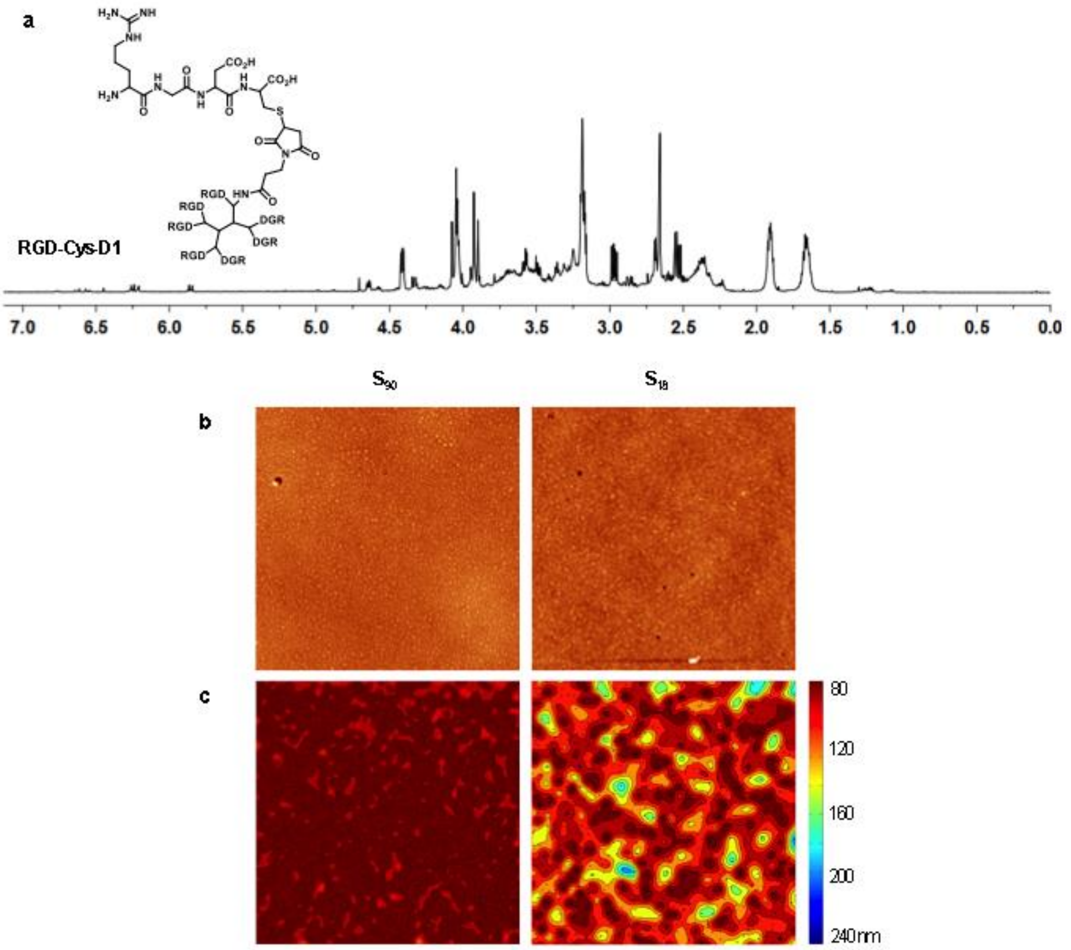

d

Nanopattern	RGD-Cys-D1 (% w/w)	Area of d_{min} < 70 nm (%)
S_{90}	2510^{9}	90±2
S_{18}	410^{9}	18±11

Figure S1. Nanopatterns of high and low local surface adhesiveness. (a) ^{1}H-NMR spectra of RGD-Cys-D1, in D_2O. Signals between 1.5 and 2.0 ppm were attributed to internal signals of the arginine (R) moiety. The remaining RGD-Cys signals overlapped those corresponding to the dendrimer (between 2.0 and 5.0 ppm). The absence of signals around 7 ppm indicates that all maleimide groups were functionalized. The spectra were acquired on a Bruker Avance 600 MHz spectrometer equipped with a 5 mm TXI inverse probe. (b) Representative atomic force microscopy (AFM) height images of the nanopatterns of RGD-functionalized dendrimers on PLLA (5x5 μm). (c) Minimum interparticle distance (d_{min}) probability contour plots, showing high-density RGD regions in dark red (d_{min} < 70 nm). Dendrimers localization is superimposed in black for clarity. (d) Percentage of area with d_{min} < 70 nm obtained for nanopatterns of high (S_{90}) and low (S_{18}) local surface adhesiveness.

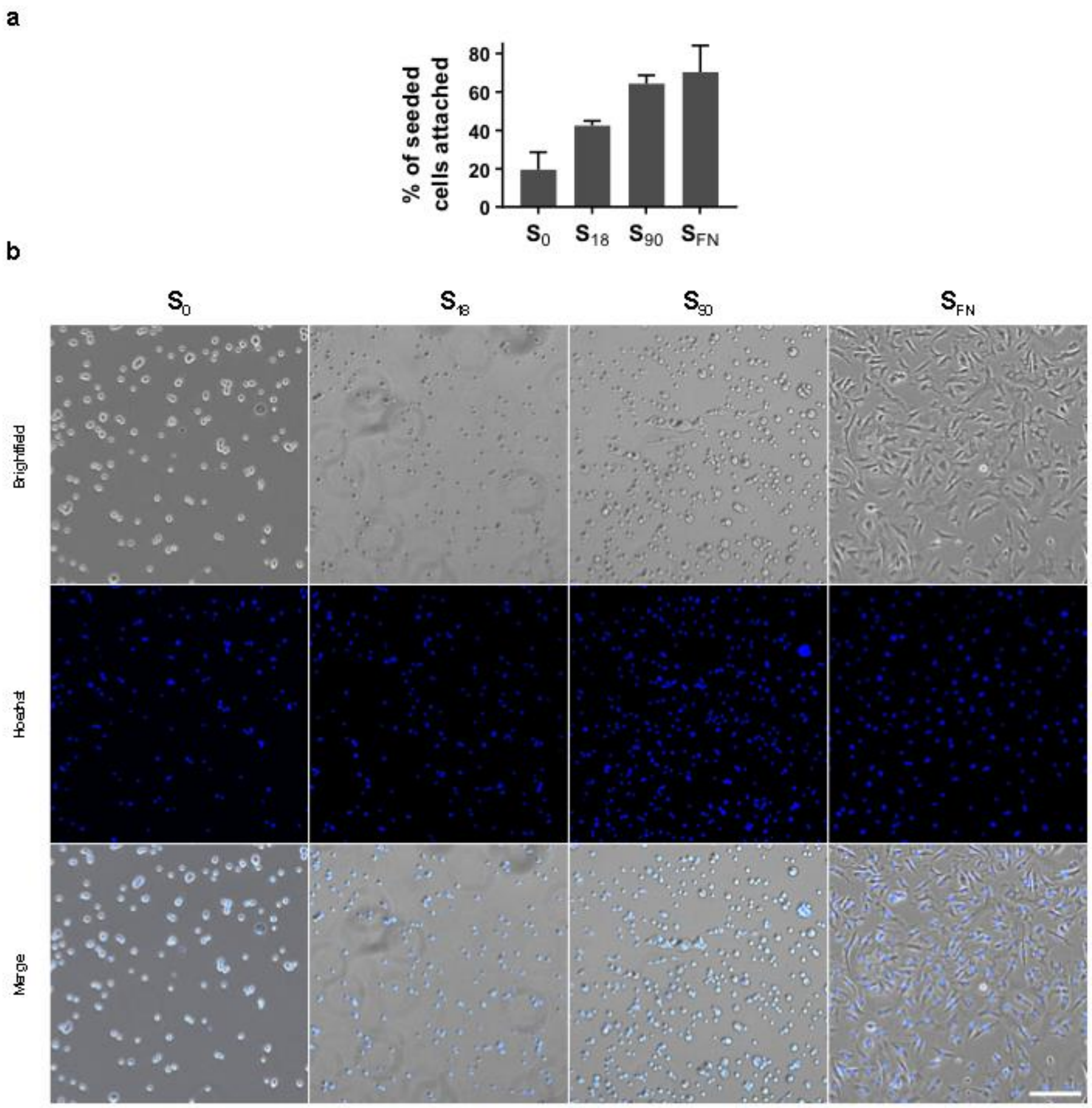

Figure S2. Initial cell adherence on the substrates. (a) Plot of the percentage of adhered cells 2 h after cell seeding. N = 2. Results are given as the mean ± SE. (b) Representative images of the adhered cells. Scale bar = 200 μm.

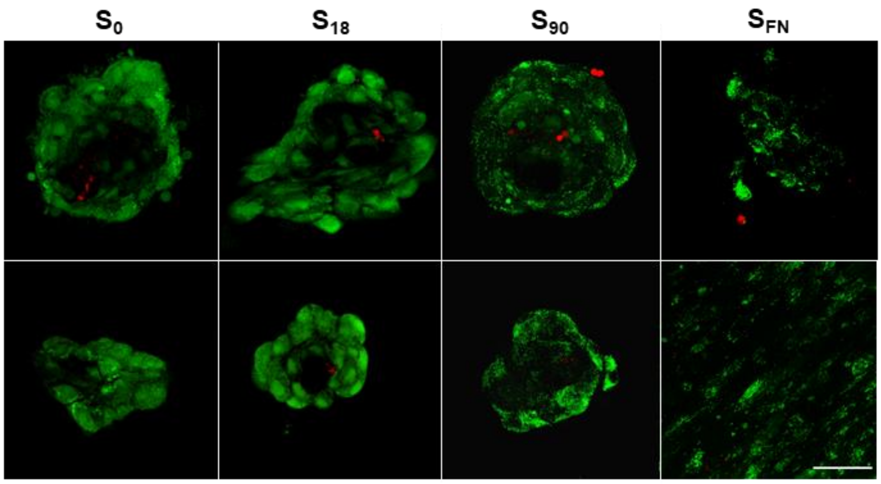

Figure S3. Cell viability assay. Samples at day 7 of chondrogenesis were stained for intracellular esterase activity, indicating live cells (green), and for nucleic acids in membrane-damaged cells (red). Two representative images are shown for each condition. Scale bar = 50 µm.

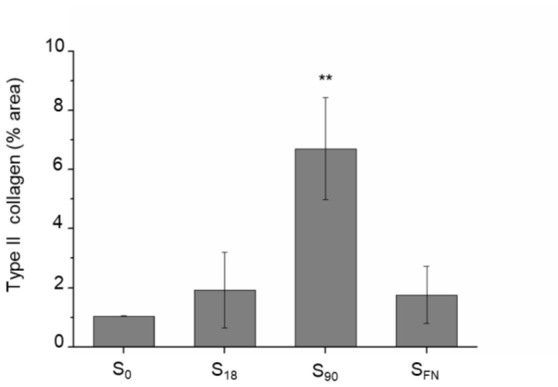

Figure S4. Type II collagen quantification. Plot of the percentage of COL2A1-stained areas in the confocal z-projections, normalized to the area of the condensate after 5 days of chondrogenic induction. N ≥ 3. Results are given as the mean ± SE. The Type II collagen expression in S_{90} nanopatterns is significantly higher than in the rest of the samples.

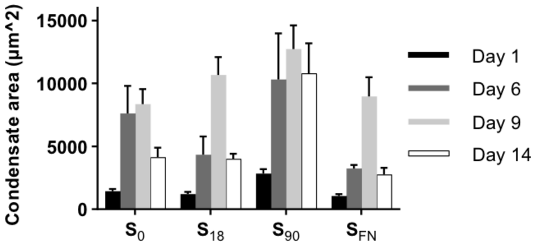

Figure S5. Condensate areas obtained from confocal image projections at different time points of chondrogenesis. N ≥ 3. Results are given as the mean ± SE. Condensates on S_{90} grow larger and are more stable in time than those on the other substrates.
